# Pharmacological articles in the German magazine *DIE ZEIT *(THE TIME)*—*content, adequacy, and comprehensibility

**DOI:** 10.1007/s00210-024-03053-3

**Published:** 2024-03-25

**Authors:** Laura Sophie Böger, Roland Seifert

**Affiliations:** Hannover Medical School, Institute of Pharmacology, Carl-Neuberg-Str. 1, 30625 Hannover, Germany

**Keywords:** *DIE ZEIT*, Drug information, Medication, Patient education, Comprehensibility, Laypeople, Experts

## Abstract

The German weekly magazine *DIE* *ZEIT* (THE TIME) reaches more than one million readers per issue, mainly from high-income social classes. Pharmacological content is frequent in *DIE ZEIT*. As it therefore reaches many people who generally have no in-depth knowledge of pharmacology, it can be assumed that *DIE ZEIT* is an important primary source of information. It should be its task to depict the drugs widely used by the population and to present them correctly and comprehensibly. This study analyzes 71 articles from 2012 to 2022 in terms of form, content, and comprehensibility. The analysis shows that in *DIE ZEIT*, drug groups largely correspond to the prescription figures and disease prevalence in Germany, with cardiovascular, neuropsychiatric, and pain medications being frequently discussed. There are deviations in the case of oncological drugs, for example, which are discussed more frequently than prescribed. New drug approvals are reported less frequently, and when they are, it is usually about the research phase. *DIE ZEIT* often reports on findings that are less than a week old and frequently quotes trustworthy experts, but no scientific sources can be found in around a quarter of the articles. A COVID-19 effect can also be identified in the years 2020 to 2022, as reporting on drugs for the treatment of coronavirus disease 2019 (COVID-19) predominated. An important point of criticism was identified with regard to comprehensibility. Less than half of the articles achieved the cut-off value for general comprehensibility specified by the Textlab analysis program, and only one article achieved the value for target group–oriented comprehensibility. This analysis confirms the problem that science communication is often too complicated and incomprehensible. It discusses the tension between the prescribed drugs and the mission of *DIE* *ZEIT* to entertain and should serve as a basis for analyzing other newspapers. Finally, we make specific suggestions how presentation of pharmacological topics in lay media can be improved in the future.

**Supplementary Information:**

The online version contains supplementary material available at 10.1007/s00210-024-03053-3.

## Introduction

The German weekly newspaper *DIE* *ZEIT* (THE TIME), founded in 1946 under Allied rule (Hess 2009, p. 75) and based in Hamburg, is published every Thursday. It is led by Giovanni di Lorenzo as editor-in-chief and is owned in equal parts by the Georg von Holtzbrinck publishing group and the DvH media group (Zeit-Online 2024, 2009). The newspaper has an edition of 600,000 copies per week (IQ-Media 2013) and reached between 1.6 million (2018) and 1.9 million (2021) readers in the years of the analysis period (2012–2022; Weidenbach 2022).

*DIE ZEIT* is classified as liberal and reports independently to enable readers to form a differentiated opinion on various topics (Hanke 2011). It is considered one of Germany’s leading media (Hess 2009, p. 76) and is aimed at an educated and high-earning readership. Seventy-one percent of readers have a university degree or at least a high school diploma and the average net household income is > € 4400 (IQ-Media 2013). The newspaper therefore has a considerable reach and serves as an important primary source of information in an educated high-income audience.

To answer the question of whether *DIE ZEIT* reports well on pharmacological topics, its important position in science communication should be emphasized in particular. Patients and laypeople have long complained that experts often communicate in a language that is difficult for outsiders to understand (Beck et al. 2021, p. 1948). Especially since the rise of the Internet, online editions of newspapers have played a particularly important role (Bamberg and Herold 2021, p. 1). They can bridge the gap between the layperson who wants to obtain general information and scientists who usually publish in peer-reviewed journals and specialized books. It is particularly important to adapt the language so that the primary goal of science communication—making science accessible to many—can be achieved. Initial analyses of this have already been carried out in some specialist areas, for example, for patient information in trauma surgery (Paul et al. 2021) or in ophthalmology (Heim et al. 2017).

To our knowledge, however, scientific analyses of pharmacological content in newspapers and magazines do not yet exist in German-speaking countries. This paper is intended to make a start in this hitherto unexplored important field by analyzing selected articles from the pharmacological content of *DIE ZEIT*.

## Material and methods

**Fig. 1:**
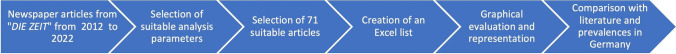
Schematic representation of the analysis procedure

Figure 1 shows the analytical procedure. First, a suitable time period was identified from which selected articles were analyzed. The last 10 years before the start of the study (mid-2012–mid-2022) were selected for this purpose. After identifying suitable analysis parameters, all 530 issues of *DIE ZEIT* were searched for articles on pharmacological topics. A total of 71 articles were selected from this period that offered sufficient pharmacological content for an analysis.

Lists in Excel were then created based on the selected parameters, which can be adequately quantified and are therefore comparable with each other as well as with existing literature. The articles were analyzed with regard to formal aspects, their content, and their comprehensibility. The latter was examined with the help of the online program Textlab. This was developed by H&H Communication Lab and the University of Hohenheim and analyzes texts according to different comprehensibility levels. It was also examined which sources were used and which authors were involved. The Excel lists of the original data are available upon request. In the next step, the results of the analysis were presented graphically and compared with existing literature as well as current figures on prevalence and prescriptions.

## Results

### Formal

#### Frequency of pharmacological content

**Fig. 2:**
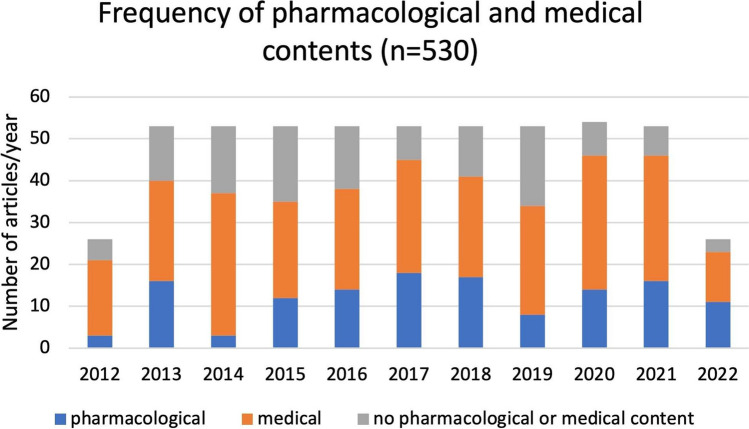
Frequency of pharmacological and medical content in all examined issues of DIE ZEIT from 2012 to 2022 (*n* = 530), shown as a bar chart

Figure 2 shows how many of the weekly issues of *DIE ZEIT* deal with medical and/or pharmacological content. In order to obtain a period of exactly 10 years, only half of the issues from 2012 and 2022 were analyzed: the last 26 issues from 2012 and the first 26 issues from 2022. In each of the years 2013 to 2021, usually 53 issues were published; in 2020, there were 54. This results in a total number of 530 issues.

Most issues contain medical and/or pharmacological articles. It is difficult to make a clear distinction between purely *medical* and purely *pharmacological* topics. Articles are considered *pharmacological* if the analysis criteria described later on are applicable. If, for example, only the names of drugs are mentioned, the article is classified thematically as medical content. Pharmacological content can be found in around a quarter of the articles. The figures range from a minimum of 3 issues in 2014 to a maximum of 13 issues in 2017.

Medical topics are present in just over half of the issues (minimum 23 issues in 2015, maximum 34 issues in 2014). The remaining issues (minimum 7 issues in 2021, maximum 19 issues in 2019) contain neither medical nor pharmacological topics.

#### Temporal relevance of the topics mentioned

Supplemental Fig. 1 shows how up-to-date the linked sources are that the authors use as a source of information for their articles. A large proportion of these can be described as extremely up-to-date. In 13 of the 71 articles, there are links to websites that were published online no more than a week before the newspaper article appeared. This basic information often refers to studies with new research findings or sales figures, but since 2020, it has also included the latest numbers of cases of coronavirus disease 2019 (Bahnsen and Grabar 2021).

In 19 of the articles, the basic information is less than 1 month old. The five articles with basic studies less than 6 months old and the one study that is less than 1 year old can also be described as up-to-date. Eleven of the articles refer to information that is already more than 1 year old. However, it is limiting that not every article uses sources. In 22 articles, either no sources are given at all, the source cannot be found, or the linked or findable source has no publication date.

#### Length of the articles

Supplemental Fig. 2 shows of how many words the individual articles consist. A maximum in the range of 1000 to 1999 words can be seen here. There are also many short articles with less than 500 words, and only two articles had more than 5000 words. On average, the articles have 1243.7 words.

### Content

#### Drugs which were featured in *DIE ZEIT*

**Fig. 3:**
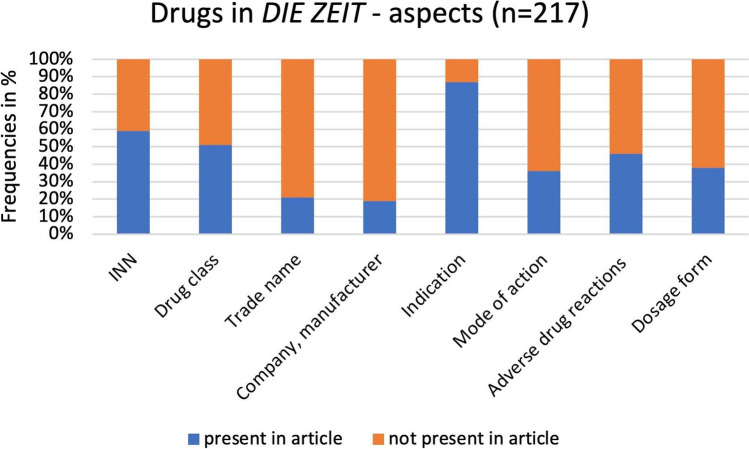
Aspects of the drugs mentioned in the articles, shown as a bar chart

An important part of this work consists of analyzing the content of *DIE ZEIT* articles. A selection of parameters was made that can be applied to the drugs mentioned to render the articles comparable with each other. A total of 217 drugs are mentioned in the 71 articles. Figure 3 shows which aspects of the drugs are presented (international non-proprietary name (INN), drug class, trade name, company/manufacturer, indication, mode of action, adverse drug reactions, and dosage form). Substantial differences can be observed. The most frequently presented aspect is the naming of indications. This occurs in 87% of cases and is probably the most important and most informative part for the average reader. In 59% of cases, defined drug names are listed and in 51% of cases, drug classes are mentioned. However, there are clear differences between the precision of the naming: sometimes, the drugs are named chemically-structurally, for example, *Opioide* (*opioids*; Albrecht 2016) or mechanistically, for example, *Neuroleptika* (*neuroleptics*; Lubahn 2020b), but sometimes, only *Herzmittel* (*cardiac agents*; Bahnsen 2017) are mentioned. The mention of trade names (21%) and companies or manufacturers (19%), entailing cost-free advertising for a product, is relatively rare but present.

The mode of action of drugs is mentioned in just 36%, adverse drug reactions in 46%, and dosage forms in 38% of the articles. With regard to the mode of action, in the vast majority of cases (88%), the correct mechanistic mode of action is also presented (for example, “Ritonavir hemmt ein Leberenzym, das unseren Wirkstoff abbaut,” Grabar 2022 [*Ritonavir inhibits a liver enzyme that breaks down our* *drug*]); in 12%, it is only the pure effect (for example, “lindern […] Schmerz und bremsen Entzündungsvorgänge,” Albrecht 2022 [*relieve […] pain and slow down inflammatory processes*]). In the case of adverse drug reactions, a distinction can be made as to whether they are specifically mentioned (87%, for example, “Knochenschmerzen, Gelenkschmerzen, Osteoporose,” Stelzer 2022 [*bone pain, joint pain, osteoporosis*]) or whether it is only stated that there are some adverse drug reactions (13%, “womöglich durch die Nebenwirkungen der Präparate,” *possibly due to the* *adverse drug reactions of the preparations*; Albrecht 2021).

The drug classes are discussed in more detail below.

#### Content analysis of organ systems, disease groups, and drug groups

For a more in-depth analysis of the content, the drugs mentioned are examined more closely in the following classifications:

- Organ systems on which they act
- Disease groups and specific diseases
- Drug groups and specific drugs

A total of 217 drugs were mentioned in the 71 articles. As some of these drugs act on several organ systems and disease groups, a total number of 272 organ systems or diseases can be determined.

**Fig. 4:**
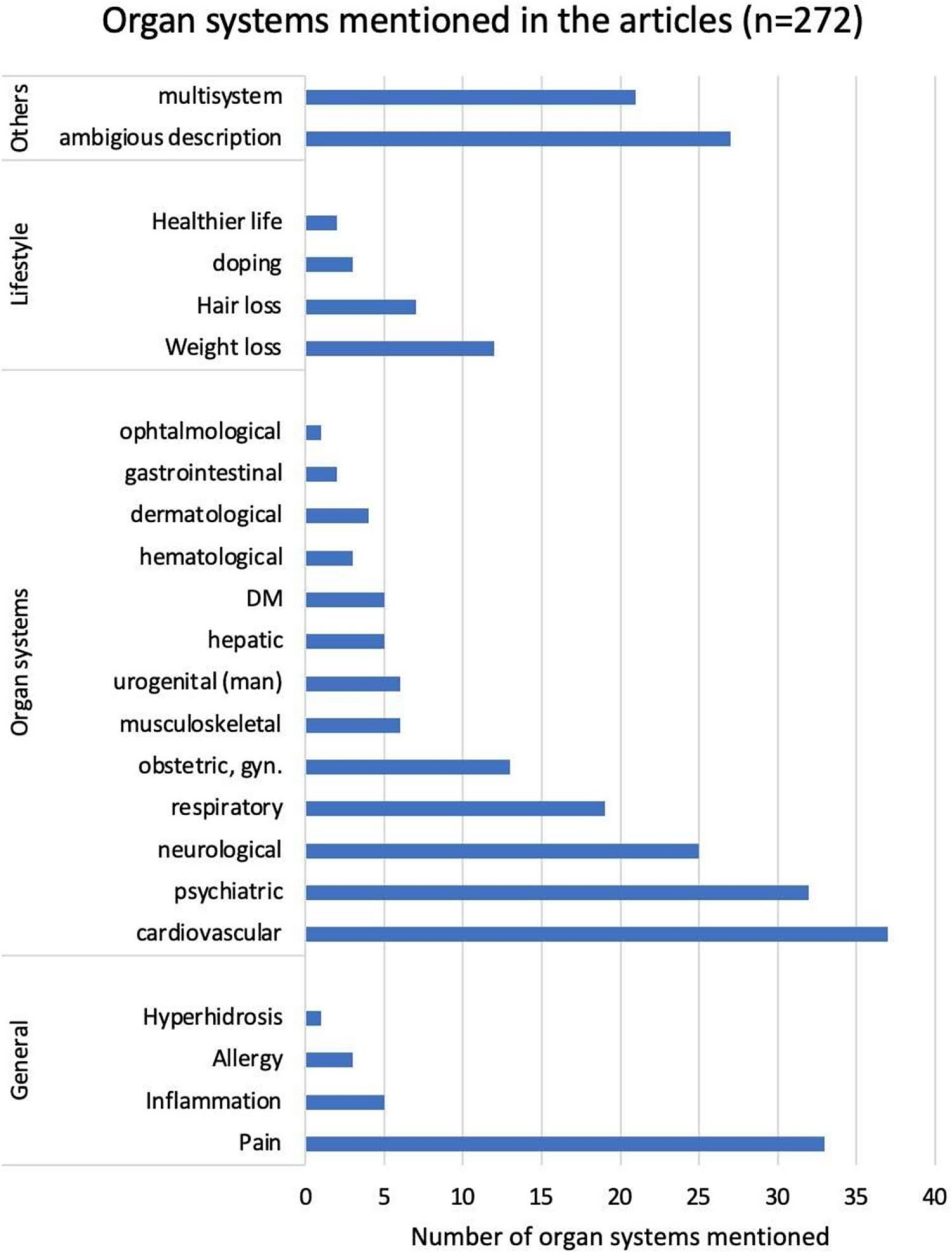
Organ systems listed in the articles with regard to the mentioned drugs, shown as a bar chart

Figure 4 shows which organ systems are affected by the drugs listed in the articles. Drugs can be categorized into four major groups:

- Drugs that have a specific effect on organ systems
- Lifestyle drugs
- Medication for general illnesses and problems
- Others (multisystemic and equivocal medications)

The latter represent a large proportion of the drugs mentioned in the articles. *Lifestyle drugs* make up only a small proportion of all drugs and relate to the areas of *weight loss*, *hair loss*, *various types of performance enhancement*, and *healthier lifestyle*. In the *general section*, drugs to combat pain make up the largest share. Medications for inflammation, allergies, and hyperhidrosis are also listed. However, most of the drugs listed directly affect organ systems, particularly the cardiovascular, neurological, psychiatric, and respiratory systems.

**Fig. 5:**
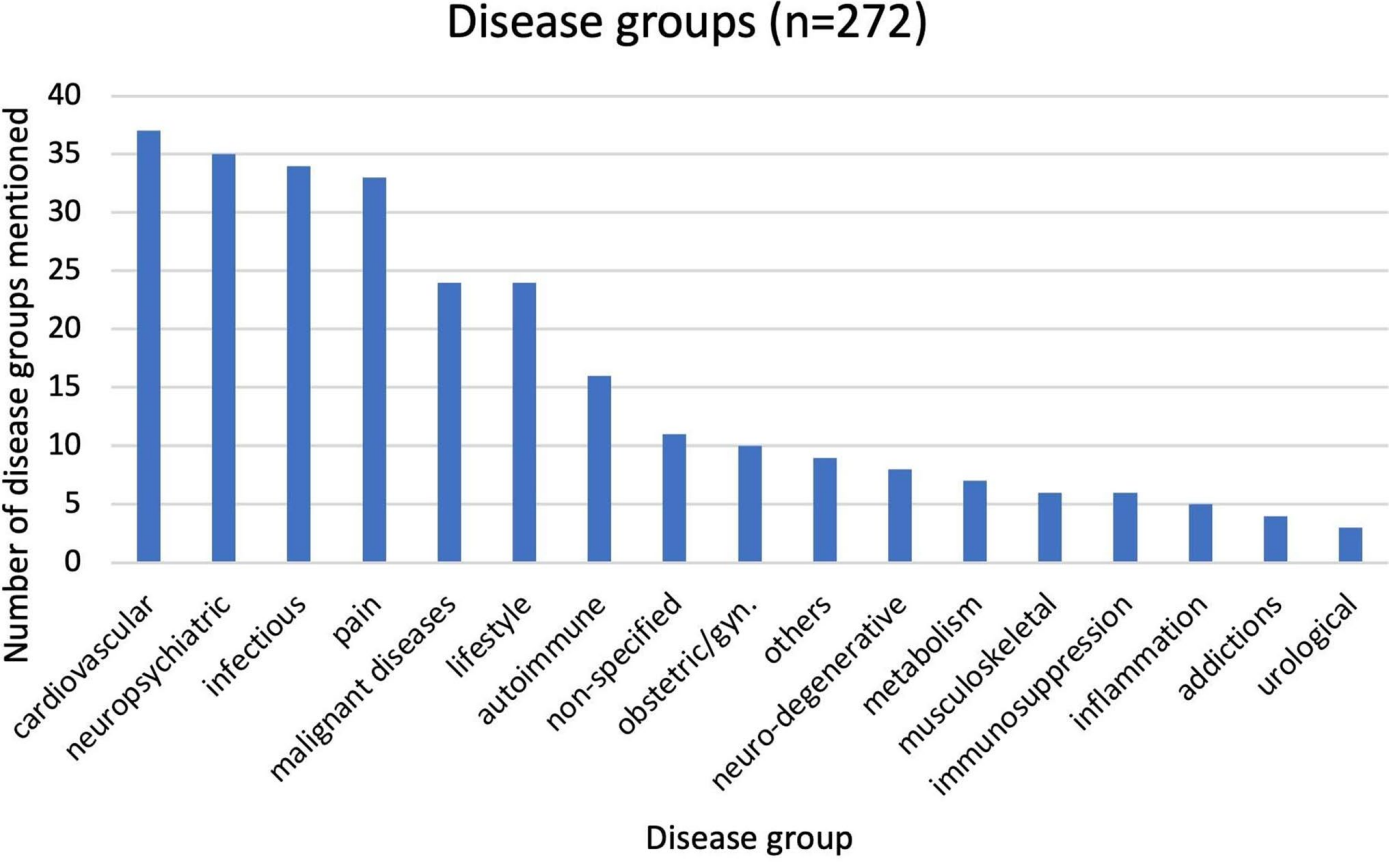
Disease groups listed in the articles, shown as a bar chart

If the drugs listed are classified according to specific disease groups, a picture shown in Fig. 5 emerges. This also shows a predominance of pain, cardiovascular, and neuropsychiatric disease groups. Many other groups in Figs. 4 and 5 overlap and have a similar frequency distribution. The individual disease groups can also be subdivided into further specific diseases. The results are available from the authors on request. In the cardiovascular diseases, for example, arterial hypertension (13 mentions) and coagulation disorders (7 mentions) dominate. The largest proportion of neuropsychiatric illnesses is depression with 13 mentions. In the area of infectious diseases, the COVID-19 disease clearly dominates (14 mentions), especially since 2020. Among the malignant diseases listed, different types are mentioned, such as breast cancer, skin cancer, and leukemia. In most cases, however, the type is not specified, with 10 mentions referring only to *malignant diseases*. In Fig. 5, the lifestyle drugs are broken down in the same way as in Fig. 4.

However, it must also be noted that not every disease group can be assigned a specific disease. *DIE ZEIT* often does not provide more precise information on the exact disease and only makes superordinate classifications itself.

**Fig. 6:**
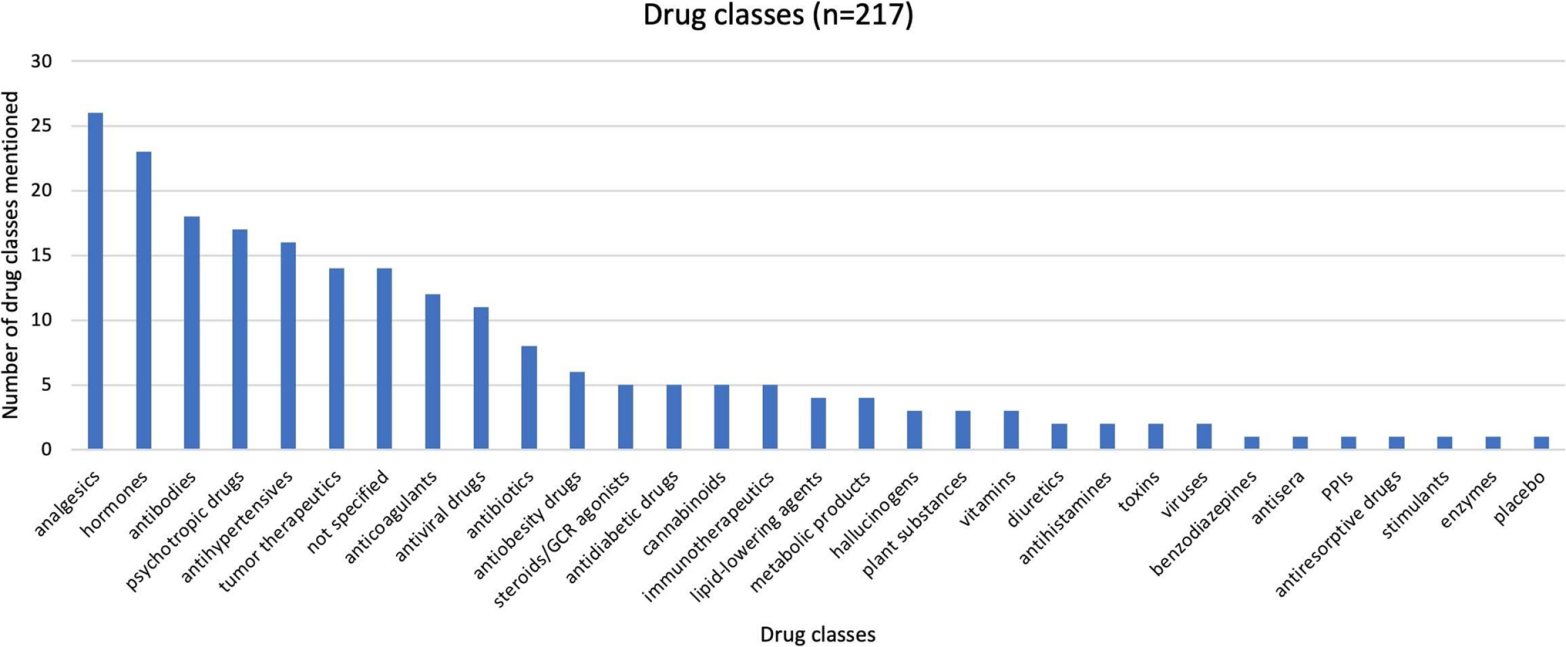
Drug classes described in the articles, shown as a bar chart

Another analysis classification examined is the assignment to broad drug groups, as shown in Fig. 6, as well as the more detailed description of the individual drugs, where possible. Similar tendencies as in Figs. 4 and 5 can also be seen here. The most frequently mentioned drugs belong to the group of analgesics, with over-the-counter, prescription drugs as well as drugs that fall under the *BtM-Gesetz* [*anesthetics law*] being mentioned. Hormones are discussed second most frequently. These are intended for various diseases, often, for example, sexual hormones for treatments of malignant diseases or as contraceptives. There is a broad spectrum of possible treatment targets for the antibodies mentioned. They are often mentioned in connection with COVID-19, rheumatic diseases, or malignant diseases. As already shown in Figs. 4 and 5, Fig. 6 also shows a large number of psychotropic drugs, antihypertensives, and tumor therapeutics.

However, some of the drugs listed cannot be clearly assigned to a drug group. This is often due to the fact that only one indication or organ system is mentioned in the corresponding articles, without it being possible to draw a clear conclusion about a group. An example of this is the sole mention of drugs for the treatment of cardiovascular diseases or chronic heart failure (CHF). Various groups of drugs could be used for treatment, such as beta-receptor blockers (correctly referred to β-adrenoceptor antagonists) (Grassi et al. 2021, p. 174), angiotensin-converting enzyme inhibitors (ACE inhibitors; Grassi et al. 2021, p. 180), or sodium-glucose transport protein 2 inhibitors (SGLT-2 inhibitors*;* Nassif et al. 2021, p. 1954).

Differences between Figs. 4, 5, and 6 are, for example, due to the fact that in some articles, certain analgesics (especially μ-opioid receptor agonists) are mentioned in connection with malignant diseases and its treatment. They are therefore included in Figs. 4 and 5 as drugs for the treatment of malignant diseases, even though they are analgesics.

### Thematic aspects of *DIE ZEIT* articles

As it is not always possible to assign a single aspect to the articles, some articles are assigned several aspects. A total of 89 aspects are covered in the 71 articles, as shown in Supplemental Fig. 3. The largest group (38 mentions) consists of articles reporting on current research and the development status of new drugs. Seventeen articles report on the political, legal, and current situation of drugs. For example, they contain information on ongoing approval procedures (Bahnsen and Albrecht 2015). Thirteen articles are personal reports by the authors. One author, for example, describes the problem of drug shortages based on her own efforts to obtain sufficient medication for her malignant disease (Stelzer 2022). Eleven articles provide general information and 9 articles present new research findings. One article provides information about the approval of a new drug.

Articles that are assigned to two aspects usually deal with the areas of *current research/development* and *policy/legal/current situation*. These topics are often interlinked and are difficult to separate.

### Comprehensibility

#### Analysis results from Textlab

The online program Textlab was used to analyze the comprehensibility of *DIE* *ZEIT* articles. This program was developed by H&H Communication Lab and the University of Hohenheim and helps users to check their texts for various parameters that are necessary for comprehensible communication (Arning and Seifert 2023, p. 1; H&H Communication Lab GmBH 2022, p. 1). Textlab analyzes texts in terms of their general and target group–oriented comprehensibility by creating scales and classifying the texts accordingly. In the case of general comprehensibility, this is the HIX (Hohenheimer Verständlichkeits Index; Hohenheim Comprehensibility Index). This assigns a text a score between 0 and 20 points based on criteria such as grammar, style, and word choice. Zero points mean poor comprehensibility, while 20 represents the best possible comprehensibility. There are four types of text to choose from in order to meet different requirements: *specialist texts*,* letters*, *online texts*, and *plain language*. The text type *letter* is chosen for the analysis, as this implies that the reader is interested in the topic but does not yet have any in-depth knowledge and would like to find out more about it. To ensure good comprehensibility, the corresponding text should achieve at least 14 out of 20 points on the HIX scale (H&H Communication Lab GmBH 2022, p. 2).

**Fig. 7:**
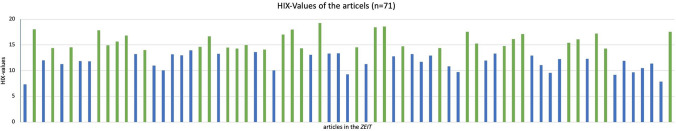
HIX value for the analyzed articles, cut-off value = 14 (articles shown in green achieve this value); shown as a column chart

When analyzing *DIE* *ZEIT* articles, the picture shown in Fig. 7 emerges with regard to the HIX.

Only 38 of the 71 articles reach the cut-off value of 14 points, while 33 are below it. On average, the articles scored 13.62 points. The least comprehensible text scored 7.35 points, while the highest scoring text scored 19.24 points. Thus, only about 53% of the texts in *DIE ZEIT* reach the threshold set by the program as minimum comprehensibility.

**Fig. 8:**
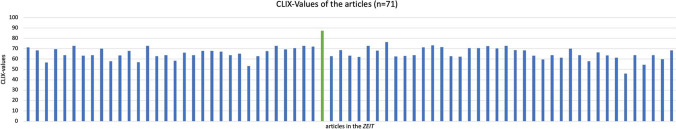
CLIX values for the analyzed articles, cut-off value = 14; shown as a column chart

Textlab offers a second analysis scale for target group–oriented comprehensibility called CLIX (Corporate Language Index). This can be customized so that users can personally select other technical language aspects in addition to the HIX parameters (H&H Communication Lab GmBH 2022, p. 4f.). Since pharmacological articles are analyzed, medical terms should not be rated as incomprehensible to a certain extent. Parameters such as too many numbers, long and complicated sentences, and a writing style that is too personal are considered unfavorable for comprehensibility. As shown in Fig. 8, a good comprehensibility seems to be even less achieved.

Only one article reaches the necessary cut-off value of 80; it scores 87 points. The article that was classified by the program as the least comprehensible only achieved 46 points. On average, the articles have a value of 65.87 points and are therefore around 14 points away from the cut-off value.

The analysis results of Textlab can be presented in more detail, as shown in Table 1.

**Table 1:** Important key figures of the linguistic analysis according to Textlab

Index	Median	Min	Max
Number of sentences	83	17	434
Word count	1075	214	5185
Number of numbers	12	0	78
Word count of longest sentence	33	20	67
Ø sentence length (in words)	12.92	9.04	16.11
Ø word length (in letters)	7.22	6.52	7.91

It is clear that the texts are difficult to understand, even apart from the content, if objective criteria are applied. The articles consist of a median of 83 sentences and are 1075 words long. The longest sentence comprises 33 words; on average, they have around 13 words. The average word length of *DIE* *ZEIT* articles is 7.22 letters. This is around 15% more than the average word length in German, which is 5.99 letters (Duden n.d.).

As shown in Table 2, *DIE ZEIT* texts are often rated as incomprehensible because they contain complicated wordings. All of the articles examined contain boxed sentences (> 2 partial sentences), words that are too long (> 16 letters), sentences with too many prepositions (> 3 prepositions), and spelling violations. With one exception, all articles also have sentences that are too long (> 20 words).

**Table 2:** Criteria of incomprehensibility, according to Textlab

Index	Cut-off value	%	Median	Min	Max
Boxed sentences	> 2 partial sentences	100	12	1	100
Sentences too long	> 20 words	98.6	11	0	48
Words too long	> 16 letters	100	16	2	100
Sentences with too many prepositions	> 3 prepositions	100	22	4	103
Misspelling		100	31	2	198

The most complicated sentence (with too many insertions) is:

Seinen Studenten, angehenden Ärzten, sagt er jetzt immer, selbst wenn sie das richtige Medikament verordnen, können sie weder sicher sein, dass es in der richtigen Dosierung eingenommen wird, noch, dass es in die richtige Körperöffnung gelangt, ja nicht einmal, ob es die richtige Person einnimmt*. *(Stock 2016);

[*He now always tells his students, future doctors, that even if they prescribe the right medication, they can neither be sure that it is taken in the right dosage, nor that it gets into* *the right orifice, nor even whether the right person is taking it*].

The longest sentence is:

Freie Radikale mit der “Smokers Infusion” zu bekämpfen kostet etwa bei einem Anbieter in Hamburg 129 Euro, die “Immunity Infusion” mit hoch dosiertem Vitamin C und Zink bekommt man für 159 Euro, und die “Superman Infusion” hält für 149 Euro neben Vitaminen und Mineralstoffen auch allerlei Aminosäuren bereit, für Menschen, die ihre “knappe Freizeit aktiv und in vollen Zügen genießen” möchten, unfreiwillige Assoziationen an beengte Bahnabteile inklusive. (Schweitzer 2020);

[*Fighting free radicals with the* *“Smokers Infusion” costs 129 euros from a provider in Hamburg, for example, the “Immunity Infusion” with high doses of vitamin C and zinc is available for 159 euros, and the “Superman Infusion” offers all kinds of amino acids in addition to vitamins and minerals for 149 euros, for people who want to “actively enjoy their limited free time to the fullest”, including involuntary associations with cramped train compartments.].*

As expected in pharmacological texts, medical terms are used in all articles, as shown in Table 3. However, other technical terms from the fields of *finance* *and* *insurance*, *legal* *German*, and *Anglicisms* can also be found.

**Table 3:** Use of technical terms in *DIE ZEIT*, according to Textlab

Index	%	Median	Min	Max
Finance and insurance	38.02	0	0	12
Legal German	29.68	0	0	6
Medical terms	100	20	1	120
Anglicisms	46.48	0	0	6

#### Sources

A total of 105 sources are used in the 71 articles. Most of the articles (30) refer to a single source, as shown in Supplemental Fig. 4. Sixteen articles have only two sources, seven articles have three, and four articles refer to four sources. A single article uses more than five sources. However, it can also be seen that 13 articles do not cite any sources or studies.

When looking at the sources used by the authors in their articles, it is noticeable that the majority of articles refer to reliable, mostly peer-reviewed journals and magazines. Examples include the *New England Journal of* *Medicine* (Albrecht 2021), the *British Medical Journal* (Simmank 2017), and the *Journal of the American Medical Association* (Drösser 2021). This information is shown in Supplemental Fig. 5. Publications from ministries and institutes (for example, the Bundesinstitut für Arzneimittel und Medizinprodukte, BfArM [*Federal Institute for Drugs and Medical Devices*]; Albrecht 2020), chambers and professional associations (for example, the Deutsche Gesellschaft für Gynäkologie und Geburtshilfe, DGGG *[German Society of Gynecology* *and Obstetrics*]; Lubahn 2020a), and companies are also frequently used. These are primarily pharmaceutical companies such as RB Pharmaceuticals (Albrecht 2017). However, health insurance companies such as the *DAK-Gesundheit* (deutsche Angestellten-Krankenkasse [*German Employees Health Insurance*]; Albrecht 2015) are also popular sources. Many articles also refer to various databases. Some sources are only insufficiently described. For example, it is not possible to find out who commissioned the “dänische Studien” (Albrecht 2018b [*Danish studies*]). Websites such as Wikipedia (Siefer 2012) or YouTube (Viciano et al. 2021) are also used.

The next step was to examine whether it is possible to trace the sources. In Supplemental Fig. 6, the 105 sources used are supplemented by the 13 articles that do not cite sources. It can be seen that many links are used in the online edition of *DIE ZEIT*; 52 sources can be accessed directly via a hyperlink in the text. Seven links lead to another *DIE* *ZEIT* article in which the primary source is linked. Eleven studies can be found through an online search and six sources can be found after a longer search on the Internet. In 29 cases, however, the source is so imprecise that it cannot be found even after intensive research. In this case, this also means that the accuracy of the article cannot be verified, as in the case of an article that refers to the source “dänische Studien” (Albrecht 2018b [*Danish studies*]). However, the figures cited refer exclusively to the online edition of *DIE ZEIT*. In the print edition, which is not part of the work, there are usually no links and therefore no references. In some exceptional cases, there is a box with references at the end of the article.

#### Experts

A total of 150 experts are cited in 71 articles, as shown in Supplemental Fig. 7. The number of experts per article varies. There are usually between one and five. Three articles cite more than five experts and in one article, more than ten are interviewed. However, there are also 24 articles in which no experts are quoted.

The distribution of cited sources and cited experts does not overlap; it is not necessarily the case that articles that do not use sources do not cite experts and vice versa.

Furthermore, the professional background of the experts mentioned are also examined, as shown in Supplemental Fig. 8. For this purpose, the professional position is used as it appeared in the article, even if the expert may hold other qualifications, professional degrees, or professional positions. The most common professional background is affiliation with universities or university hospitals, with 64 of the experts holding such a position. Thirty-one experts work for independent authorities, institutions, or societies. Eleven are employed by companies, mostly pharmaceutical companies, and nine work in politics or political institutions. Of the 35 experts who are named without belonging to a group, 19 are doctors, seven are researchers or study directors, three are psychologists, three are pharmacists, one is a lawyer, one is a sociologist, and one is an author. These individuals may also hold other positions in institutions or similar facilities. However, this is not taken into account here and only the professions mentioned in the article are listed.

#### Authors

As shown in Supplemental Fig. 9, a total of 85 authors are involved in the 71 articles. Most of the articles (62) are written by a single author. Six articles are written by two authors, and three articles by three authors. No article has four authors, and one article has five authors.

As can be seen from Supplemental Fig. 10, approximately a third (32%) of the articles were written by a woman, and about two-thirds (68%) of the texts were written by a man. However, as some authors wrote several articles, this results in a total number of only 38 different authors. Looking at the gender distribution in this way results in a rather balanced distribution, as shown in Supplemental Fig. 11. Fifty-three percent of the authors are male; 47% are female.

In order to assess the professional accuracy of the articles, the professional backgrounds of the authors are also examined, as published by *DIE ZEIT* itself (see Supplemental Fig. 12). Eleven authors are named *author*, *journalist*, or *editor* without any further professional title. Seven are doctors or medical students. A further six authors have a background in STEM fields, particularly biology and physics. Six authors work in the humanities, and one psychologist and one graphic designer are also involved. Six authors do not provide any professional details.

In a final step, the employment status of the authors is examined in order to assess whether one employment group is given priority for publication. Of the authors, 20 are employed by *DIE ZEIT*, 13 are freelancers, and for five authors, this cannot be determined (Supplemental Fig. 13). As expected, slightly more articles are published by employed authors than by other authors.

## Discussion

**Fig. 9:**
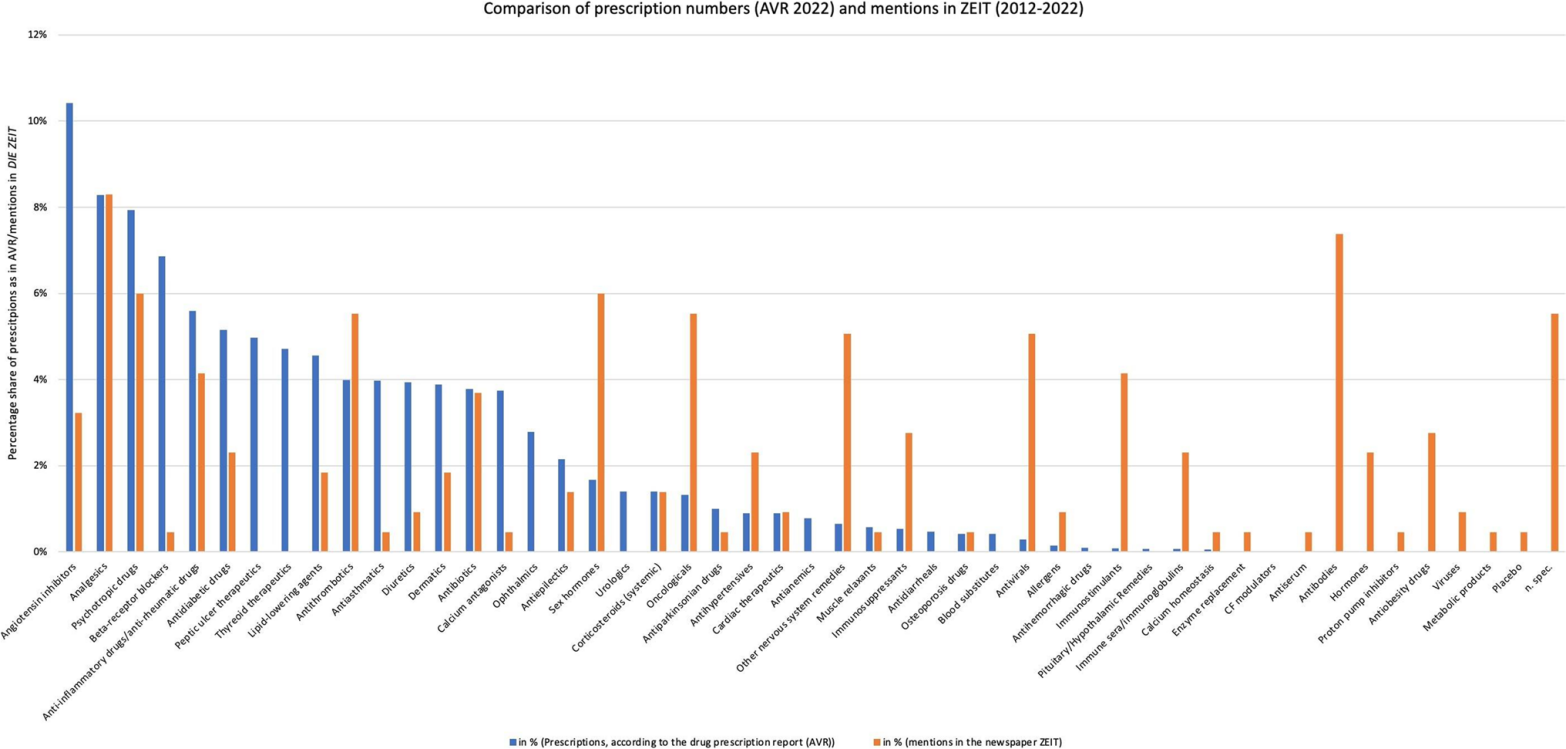
Comparison of percentage prescription numbers according to AVR 2022 and percentage mentions in DIE ZEIT, shown as a bar chart

To find out whether *DIE* *ZEIT* is now making a good contribution to informing the public, the results presented are compared with disease prevalence in Germany and other literature. When comparing the mentions of *DIE ZEIT* with the prescriptions in Germany as shown in the Arzneiverordnungsreport ([*Drug Prescription Report*], AVR; Ludwig et al. 2023, p. 10f.), the picture is mixed (Fig. 9). The most frequently prescribed drug group in Germany, angiotensin inhibitors, is mentioned less than half as often in the percentage comparison. The mentions of analgesics are almost exactly the same, and psychotropic drugs and anti-inflammatory/antirheumatic drugs also show no major difference. In Germany, beta-receptor blockers and antidiabetic drugs are also frequently prescribed, but they are underrepresented in *DIE ZEIT*. Peptic ulcer and thyroid therapeutics, which are also frequently prescribed, are not mentioned in *DIE* *ZEIT.* In contrast, antithrombotics, oncologicals, other antihypertensives, other drugs for the nervous system, immunosuppressants, antivirals, allergens, immunostimulants, and immune sera/immunoglobulins are overrepresented in *DIE ZEIT*. This is due to many different factors.

The increased number of articles on antivirals can be attributed to the COVID-19 effect of 2020 to 2022. A broad part of the population was interested in ways to combat the viral disease, and *DIE* *ZEIT* thus fulfilled its duty to inform (Meckel 2008, p. 261; Hess 2009, p. 86). There was a lot of research and new findings on the topic that was written about during these years. In the case of oncologicals, immunostimulants, and immune sera/immunoglobulins, their disproportionate mention is explained by the increased research and the many possibilities for malignant disease and immune treatments in the future. New drugs in these areas are among the most expensive of all (Kansteiner et al. 2023), so *DIE* *ZEIT* can also see a need for information here.

Some drug classes mentioned in *DIE* *ZEIT* cannot be assigned to the classifications of the Drug Prescription Report. These include antibodies, unspecified hormones, and antiobesity drugs. This is usually due to imprecise naming in *DIE ZEIT*, as is the case with antibody drugs, for example. Others, such as antiobesity drugs, are prescribed too rarely to be listed in the Drug Prescription Report (Ludwig et al. 2023, p. 10f.).

Another considerable proportion of the drugs mentioned in *DIE* *ZEIT* are described too imprecisely to be assigned to a drug class. This means that they cannot be compared with the actual prescriptions.

Another interesting point of analysis is to check whether the disease groups in the *DIE* *ZEIT* articles, as shown in Fig. 5, correspond to the actual prevalence figures for diseases in Germany. Here, too, it can be seen that the figures correspond to some extent.

The disease group most frequently mentioned in *DIE* *ZEIT* is cardiovascular disease. In Germany, the prevalence of coronary heart disease is around 6% (Heidemann et al. 2021, p. 10), and cardiovascular diseases also represent the largest group of causes of death at 40% (Robert Koch-Institut 2023). Neuropsychiatric illnesses such as depression are also often described in *DIE ZEIT* and are also widespread in the population. In a health survey in Germany, 8.8% of interviewees stated when asked that they had experienced depressive symptoms in the two weeks prior to the survey (Heidemann et al. 2021, p. 9). *DIE* *ZEIT* cites infectious diseases as the third most common group of illnesses. These were mostly COVID-19 infections. This also corresponds to the frequency in the population, with over 38 million infections in Germany by January 2024, including multiple infections of individual citizens (Siekmann 2024). Pain is widespread both in *DIE ZEIT* (fourth most common group) and in the population: 60% of respondents to a study by the Statistisches Bundesamt [*Federal Statistical Office*] suffered from pain in the 12 months prior to the survey (head, back, joint, abdomen, muscle, tooth, other; Statista 2021). Articles about malignant diseases drugs are also frequently found in *DIE ZEIT*, and although only 1.9% of people in Germany were diagnosed with malignant diseases in the 5 years prior to a 2019/20 study (Robert Koch-Institut, Gesellschaft der epidemiologischen Krebsregister in Deutschland e.V. 2023, p.19; Statistisches Bundesamt n.d.), these articles are frequent, as the development of these drugs often attracts great interest. This is partly because they are usually expensive and partly because malignant diseases, as a supposedly incurable diseases, are particularly emotionally charged.

Allergies are widespread in the population (up to 30%; Heidemann et al. 2021, p. 11), urological sexual problems (33.4% of men and 45.7% of women; Briken et al. 2020, p. 654), and musculoskeletal diseases such as osteoarthritis at 17.1% (Heidemann et al. 2021, p. 12). There are also many young women who take oral contraceptives (35–45%; Janson 2021); these four topics are also frequently represented in *DIE ZEIT*.

It should also be noted that the prevalence of chronic diseases increases significantly with age and therefore, more people in the age group of *DIE ZEIT* readers may be affected by the respective diseases than the average of the survey.

*DIE ZEIT* publishes articles particularly frequently on drugs that are about to be launched on the market, are currently in the approval process, or have recently been approved in Germany. As Table 4 shows, these thematic mentions in *DIE ZEIT* can be related to the time of approval, but some are also reported much earlier or later, usually in connection with new application possibilities such as repurposing (Specht and Seifert 2023, p. 1) or new or other indications.

**Table 4:** Comparison of newly introduced medications according to AVR with mentions in *ZEIT*

Drug	Arzneiverordnungsreport (AVR) [*Drug Prescription Report*]	Indication Drug Prescription Report	Introduction	*DIE ZEIT*	Indication *DIE ZEIT*
Molnupiravir	AVR 2023 (Ludwig et al. 2024, p. 36)	COVID-19	03/01/2022	Bahnsen and Grabar 2021 Albrecht 2021	COVID-19 COVID-19
Remdesivir	AVR 2022 (Ludwig et al. 2023, p. 32)	COVID-19	01/06/2021	Viciano et al. 2021 Albrecht 2021 Bahnsen et al. 2020 Buchter et al. 2020	COVID-19, Ebola COVID-19, Ebola COVID-19, Ebola, Hep. C COVID-19, Ebola, Hep. C
Fenfluramine	AVR 2022 (Ludwig et al. 2023, p. 33)	Dravet syndrome	01/02/2021	Schäfer 2013	Weight loss
Erenumab	AVR 2019 (Schwabe et al. 2019, p. 67)	Migraine prophylaxis	01/11/2018	Albrecht 2018a	Malignant diseases, migraine
Avelumab	AVR 2018 (Schwabe et al. 2018, p. 55)	Merkel cell carcinoma	15/10/2017	Maier 2017	Skin cancer
Sarilumab	AVR 2018 (Schwabe et al. 2018, p. 56)	Rheumatoid arthritis	15/06/2017	Albrecht 2021	Rheumatoid arthritis, immunosuppression
Dimethyl fumarate	AVR 2015 (Schwabe and Paffrath 2015, p. 41)	Multiple sclerosis	01/03/2014	Grabar 2013	Multiple sclerosis, psoriasis
Peginterferon beta-1a	AVR 2015 (Schwabe and Paffrath 2015, p. 42)	Multiple sclerosis	01/09/2014	Daum and Grabar 2013	Multiple sclerosis
Teriflunomide	AVR 2014 (Schwabe and Paffrath 2014, p. 51)	Multiple sclerosis	01/10/2013	Grabar 2013	Multiple sclerosis, rheumatic disease

Although molnupiravir, a drug for oral treatment of COVID-19 in non-hospitalized patients, was only introduced in Germany at the beginning of 2022, it was already covered in *DIE* *ZEIT* in 2021. Remdesivir is mentioned once after its introduction to treat patients with COVID-19 and three times before that. The serotonin-releasing agent fenfluramine, which was introduced in 2021 for the indication Dravet syndrome, was already mentioned once in *DIE ZEIT* in 2013 as a weight loss medication. In 2018, *DIE* *ZEIT* reported on the human anti-CGRP receptor monoclonal antibody erenumab, which can be used in migraine prophylaxis, even before its launch in November 2018. The Drug Prescription Report 2018 (market launches in 2017) mentions two antibodies, avelumab and sarilumab. Avelumab is discussed in *DIE ZEIT* even before its launch in Germany; sarilumab only 3 years after its launch. Although dimethyl fumarate and peginterferon beta-1a were only introduced in Germany in 2014 as a prescription medicine used to treat relapsing forms of multiple sclerosis, *DIE ZEIT* already reported on them in 2013. *DIE* *ZEIT* reported on teriflunomide just a few weeks before it was approved as a multiple sclerosis drug in Germany. The Drug Prescription Reports from 2021, 2020, 2017, 2016, and 2013 do not list any newly introduced drugs that were also covered in *DIE ZEIT*.

This shows that *DIE* *ZEIT* rarely reports on newly launched drugs, but rather on drugs that have already been approved or on developments leading up to approval.

If one compares the comprehensibility of the pharmacological content of *DIE ZEIT* with other medical texts, a similar picture emerges. *DIE* *ZEIT* only achieves the minimum comprehensibility of 14 out of 20 points on the HIX scale in around 54% of cases. The mean value is 13.62 points. Other studies that examine specialist texts using the Textlab analysis program come to similar conclusions. A study from 2023, which analyzed the comprehensibility of package inserts, found an average comprehensibility of 10.2 points (Arning and Seifert 2023, p. 1). An analysis of information materials on ophthalmological diseases provided on the websites of university hospitals shows an average of 7.91 points (Heim et al. 2017, p. 453). Another study examines patient information on trauma surgery injuries and their treatment; here, the authors only determined a HIX score of 4.1 points (Paul et al. 2021, p. 188). In a direct comparison with the aforementioned works, *DIE* *ZEIT* still achieves the highest score and thus proves to be the most comprehensible. This is possibly due to the fact that, for example, a patient information is written by very knowledgeable people who are not necessarily able to judge the extent to which the content needs to be expressed in a way that is easier for laypeople to understand. *DIE* *ZEIT* authors are less likely to be physicians, but are instead members of the social sciences, for example, and are therefore better able to assess comprehensibility.

In addition, unlike patient information, *DIE* *ZEIT* is not publicly accessible to everyone, but requires a paid subscription or at least the purchase of a single issue. This and the assumption that the reader, if he is interested in a pharmacological article, must already be somewhat educated, leads to more sophisticated writing in *DIE ZEIT*. This means that *DIE* *ZEIT* does a good job in terms of readership compared to the other analyses.

The analysis and comparison results described above can be summarized as shown in Table 5. While DIE ZEIT is not perfect, it receives good ratings in 7 out of 11 categories, mediocre ratings in 3 categories, and just one poor rating. The deficiencies identified are very specific and can be readily addressed and improved.

**Table 5:**
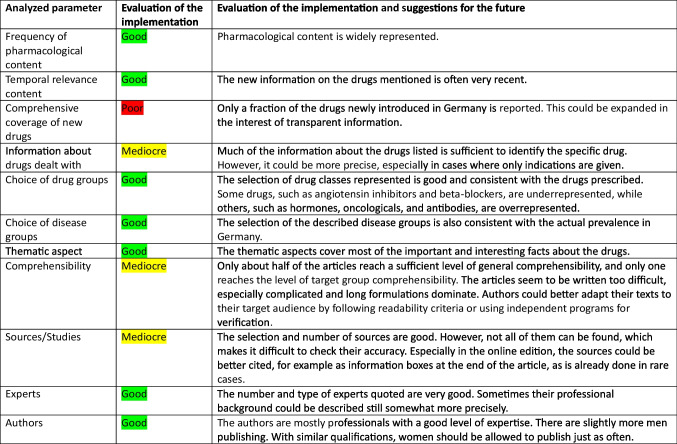
Summary of analyzed parameters in *DIE ZEIT* and their evaluations and suggestions for improvement

### Limitations

It should be noted that Textlab is an automated online program. Readers’ comprehension is very individual and depends on many criteria such as interests, background knowledge, and existing vocabulary (Elleman and Oslund 2019, p. 4). Whether articles are comprehensible to the reader should therefore not be judged solely on the basis of Textlab’s analysis results. It is also only possible to choose between three text types to which the comprehensibility should be adapted. This means that it is not possible to specifically adapt the selected text type *letter* to *DIE* *ZEIT* and its readership.

Furthermore, *DIE ZEIT* does not fulfill the requirements of a scientific journal. Its task as a weekly magazine is to provide its readership with broad and correct information as well as a point out issues that the public might not yet be aware of (Hess 2009, p. 86), but not in the technical depth that scientific journals do. Therefore, topics like different names for the drugs or not mentioning all adverse drug reactions are not so important. The use of sources such as Wikipedia or YouTube is also more feasible in *DIE ZEIT* than in scientific articles.

Similarly, a complete comparison of the topics described in *DIE ZEIT* and the medical reality is only possible to a limited extent. Not all drugs are clearly named in *DIE ZEIT* and cannot be assigned to medicines from the Drug Prescription Report. However, in the majority of cases where this is possible, the thematic coverage is good.

The topic covered in this paper is novel and has not been examined before. Thus, no extrapolations from this analysis to other German news magazines such as DER SPIEGEL (The Mirror) or STERN (Star) can be made.

### Future studies

It is advisable for the authors of *DIE ZEIT* to use readability criteria such as the HIX as a guide when writing an article in the future or to check their texts with a program such as Textlab. In addition, journalists writing articles on pharmacological topics may wish to consult the topic checklist provided in this paper (Table 5). Lastly, it will be important to analyze other magazines and newspapers according to our analysis scheme to obtain a more complete picture on the presentation of pharmacological content in the lay press.

## Take-home messages

- *DIE ZEIT* publishes pharmacological content in up to a quarter of its issues, reaching over 1.6 million readers every week.
- It deals with very topical issues that are often less than a week or a month old.
- The sources and experts are mostly of high quality, but some of them are not sufficiently cited to be found.
- For the most part, *DIE* *ZEIT* names drugs and drug classes precisely, provides a lot of information on indications, and sufficiently describes effects, adverse drug reactions, and dosage forms.
- The drugs mentioned in *DIE ZEIT* largely correspond to the drugs prescribed in Germany and the prevalence of diseases and affected organ systems. Hormone and antibody therapies, oncologicals, and immunostimulants are mentioned more frequently, whereas angiotensin inhibitors and beta-receptor blockers are clearly underrepresented.
- A clear COVID-19-effect can be seen in the area of antivirals.
- The comprehensibility of the articles can be classified as rather inadequate, which corresponds to the general picture of texts on patient education. However, *DIE* *ZEIT* performs better than other texts that have already been analyzed.
- There are possibilities to make patient information more comprehensive and easier to understand.

## Supplementary Information

Below is the link to the electronic supplementary material.

Supplementary file1 (DOCX 820 KB)

## Data Availability

All source data for this project are available from the authors upon reasonable request.

## Abbreviations

ACE: Angiotensin-converting enzyme
AVR: Arzneiverordnungsreport (Drug Prescription Report)
BfArM: Bundesinstitut für Arzneimittel und Medizinprodukte (Federal Institute for Drugs and Medical Devices)
BtM: Betäubungsmittel (anesthetics)
CHF: Chronic heart failure
CLIX: Corporate Language Index
DAK: Deutsche Angestellten-Krankenkasse (German Employees Health Insurance)
DGGG: Deutsche Gesellschaft für Gynäkologie und Geburtshilfe e. V. (German Society of Gynecology and Obstetrics)
HIX: Hohenheimer Verständlichkeits Index (Hohenheim Comprehensibility Index)
INN: International non-proprietary name
PPIs: Proton pump inhibitors
SGLT-2: Sodium-glucose transport protein 2
STEM: Science, technology, engineering, mathematics
