# Supplementary material for: Pharmacological articles in the German magazine DIE ZEIT (THE TIME)—content, adequacy, and comprehensibility
Source: Naunyn Schmiedebergs Arch Pharmacol. 2024 Mar 25;397(9):6763–78. doi: 10.1007/s00210-024-03053-3 (PMC11422248; doi:10.1007/s00210-024-03053-3)
Supplement: Supplementary file 1 — Supplementary file1 (DOCX 820 KB) [file 210_2024_3053_MOESM1_ESM.docx]

**Supplemental Figures**

**Pharmacological articles in the German magazine *DIE* *ZEIT
(THE TIME)* – content, adequacy, and comprehensibility**

**Laura Sophie Böger and Roland Seifert**

Correspondence: Roland Seifert, Hannover Medical School, Institute of Pharmacology, Carl-Neuberg-Str. 1, D-30625 Hannover, Germany

seifert.roland@mh-hannover.de


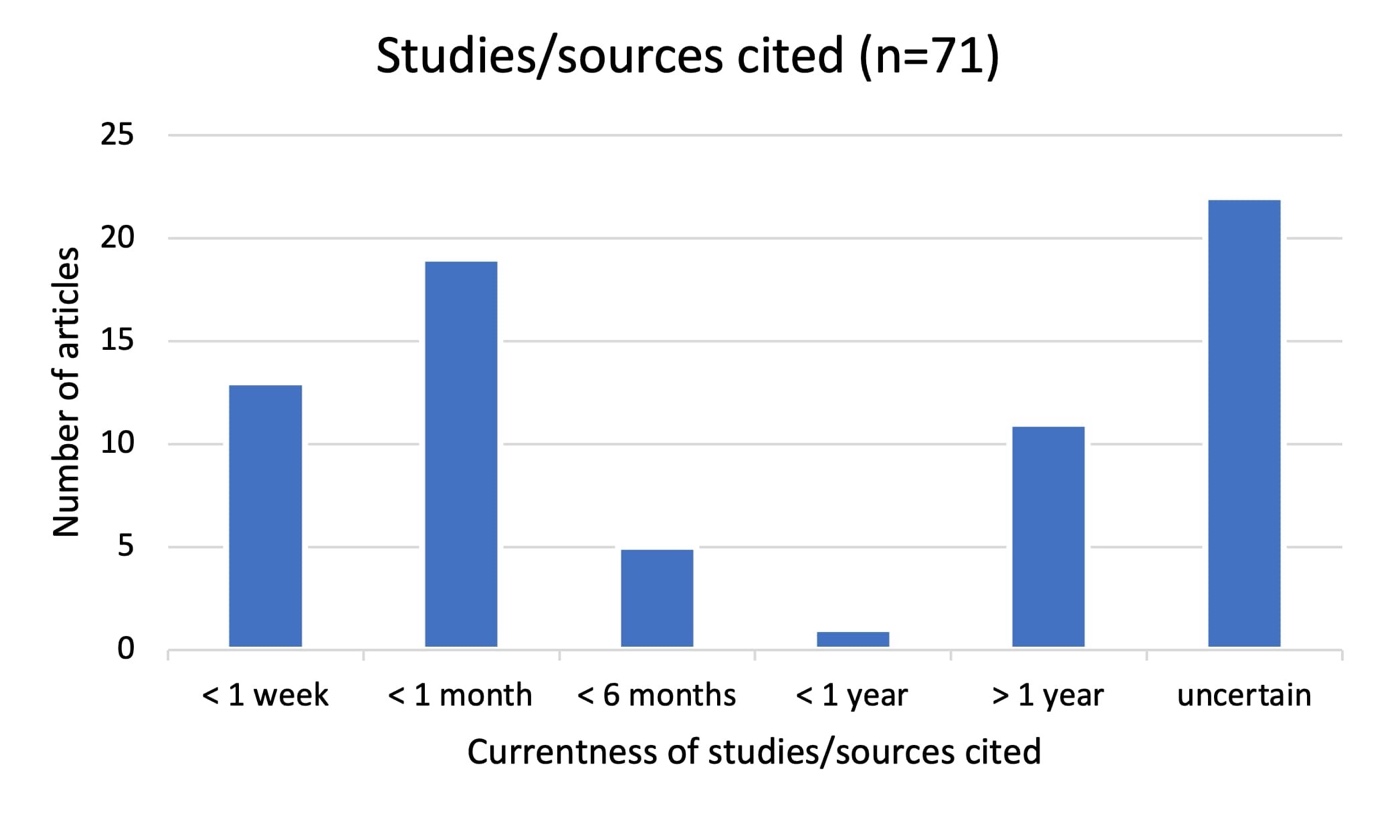


Supplemental Figure 1: Currentness of the studies and sources cited that served as the basis for the articles, shown as a bar chart


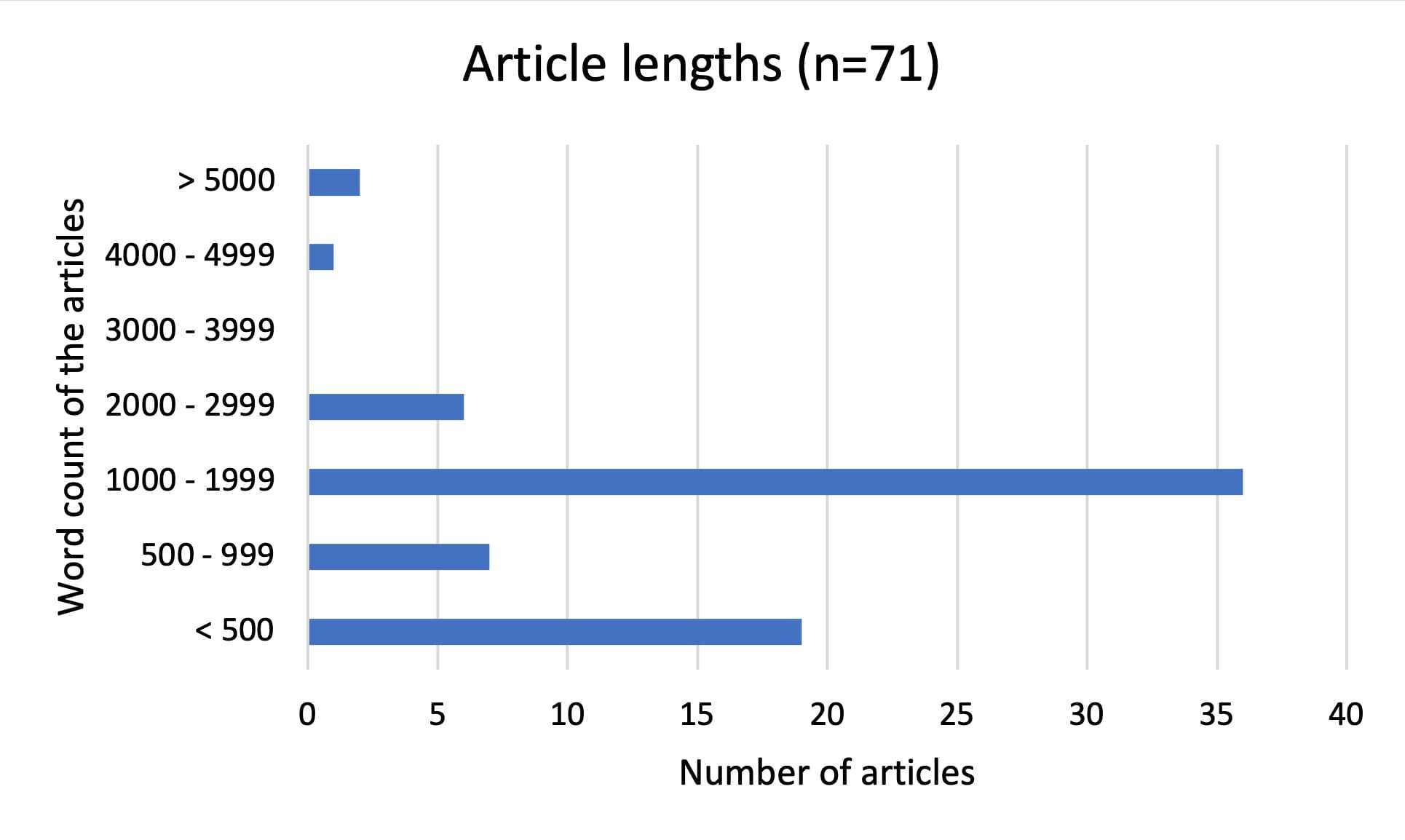


Supplemental Figure 2: Length of articles including titles and subtitles, excl. author and publication date, shown as a bar chart


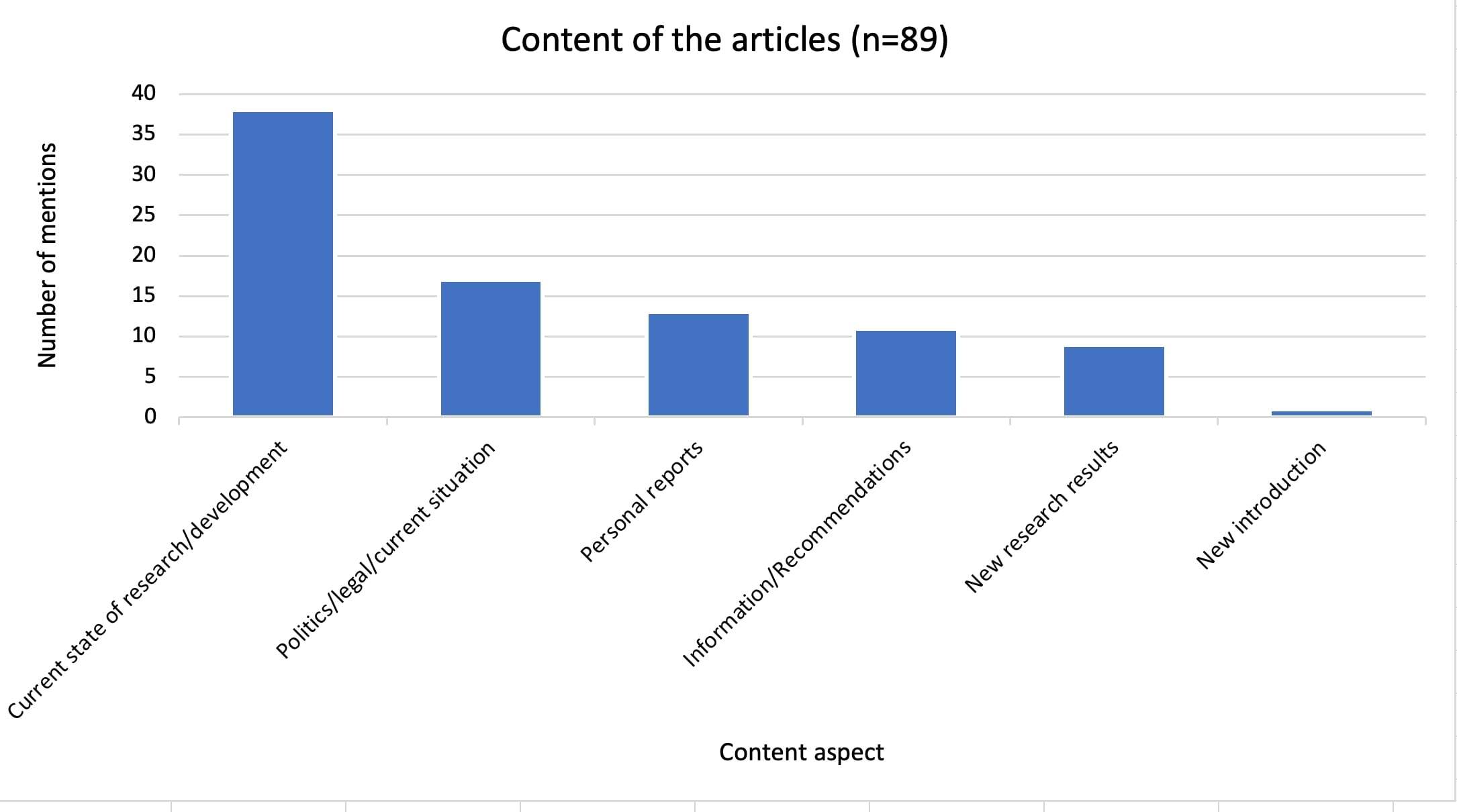


Supplemental Figure 3: Thematic aspects of the articles, shown as a bar chart


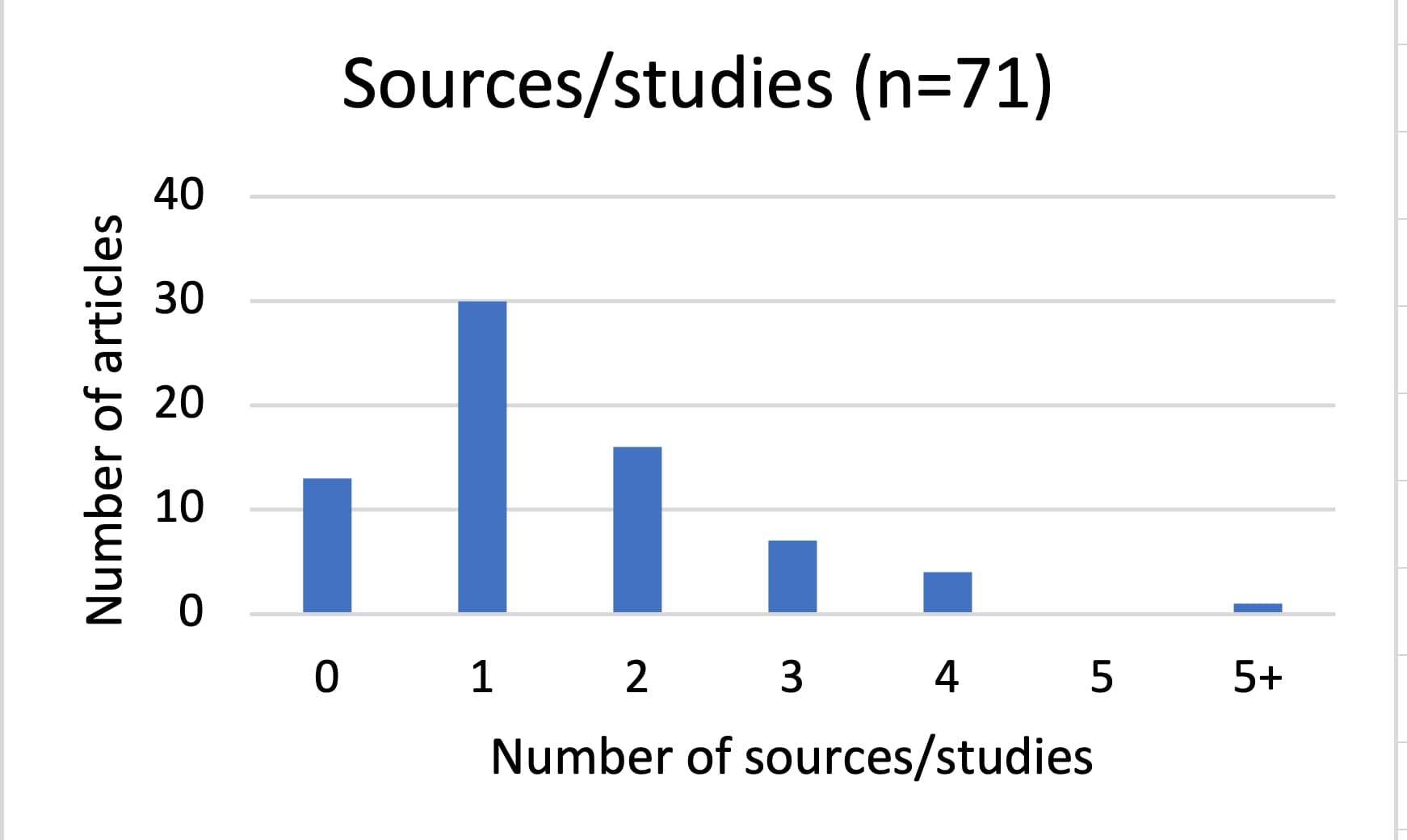


Supplemental Figure 4: Number of sources/studies used per article, shown as a bar chart


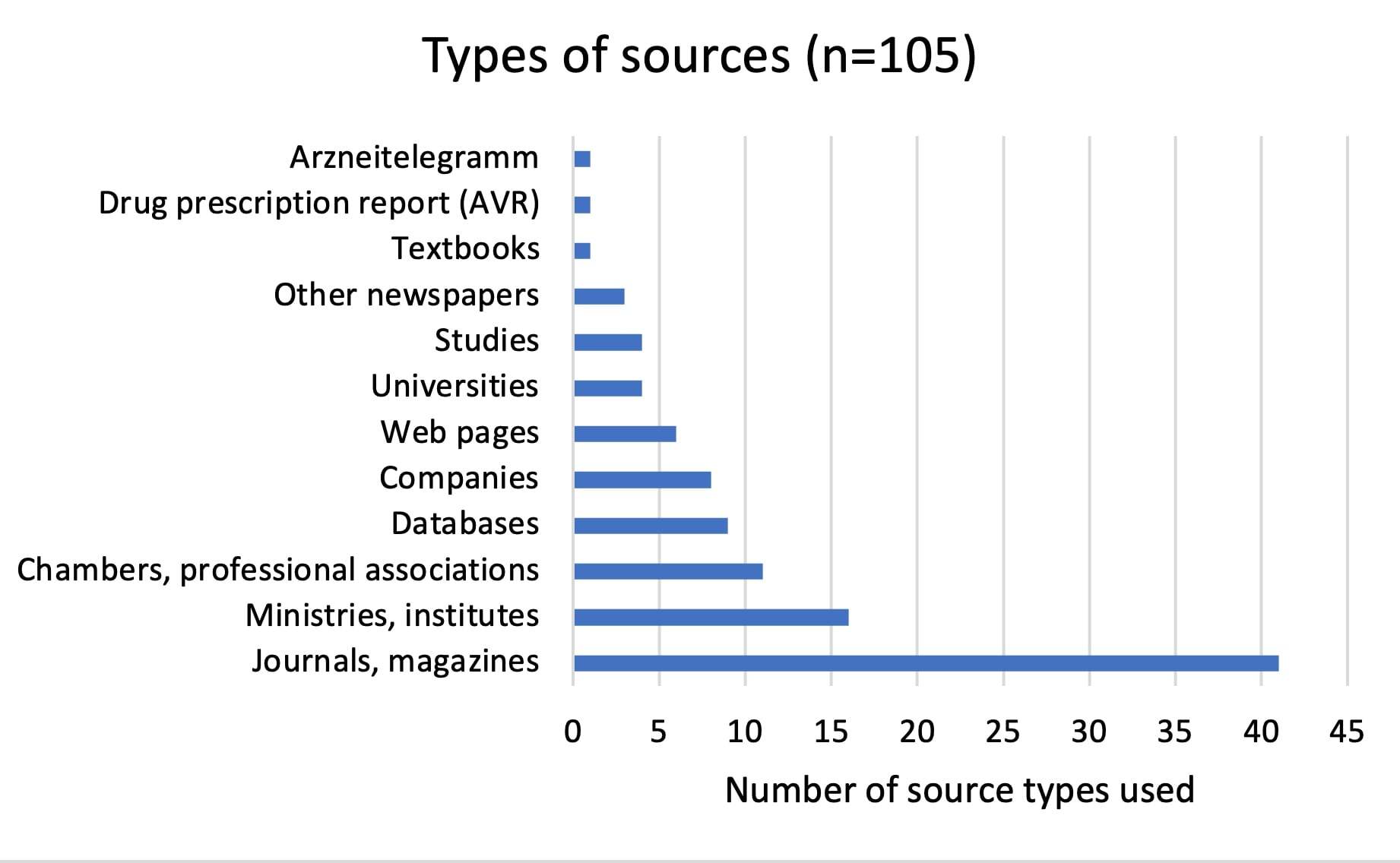


Supplemental Figure 5: Types of sources used in the articles, shown as a bar chart


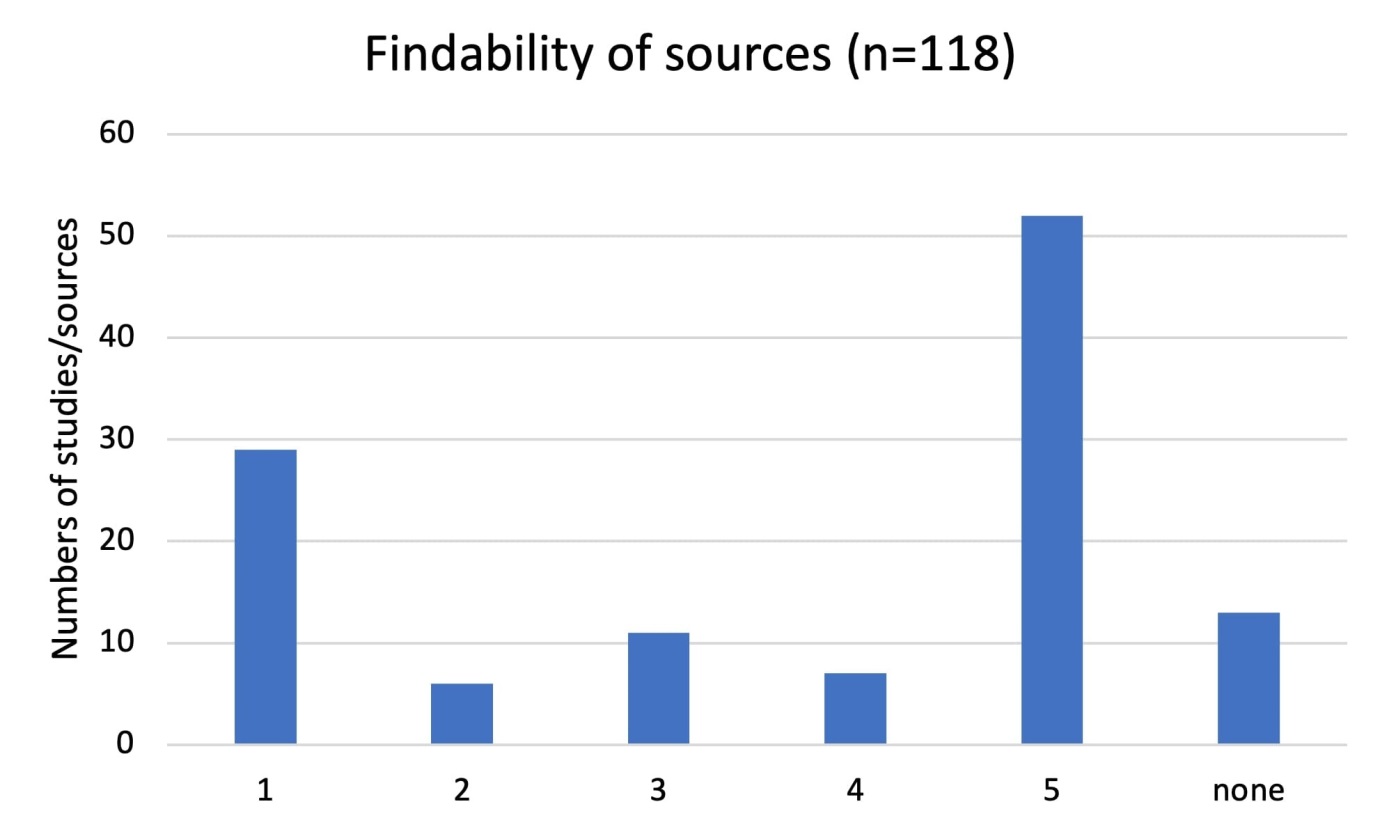


Supplemental Figure 6: Findability of sources using a points system. 13 articles had no source. 1=not found; 2=found after lengthy own research; 3=can be found with own research; 4=another ZEIT article linked in which the study is linked, 5=directly linked; shown as a column chart.


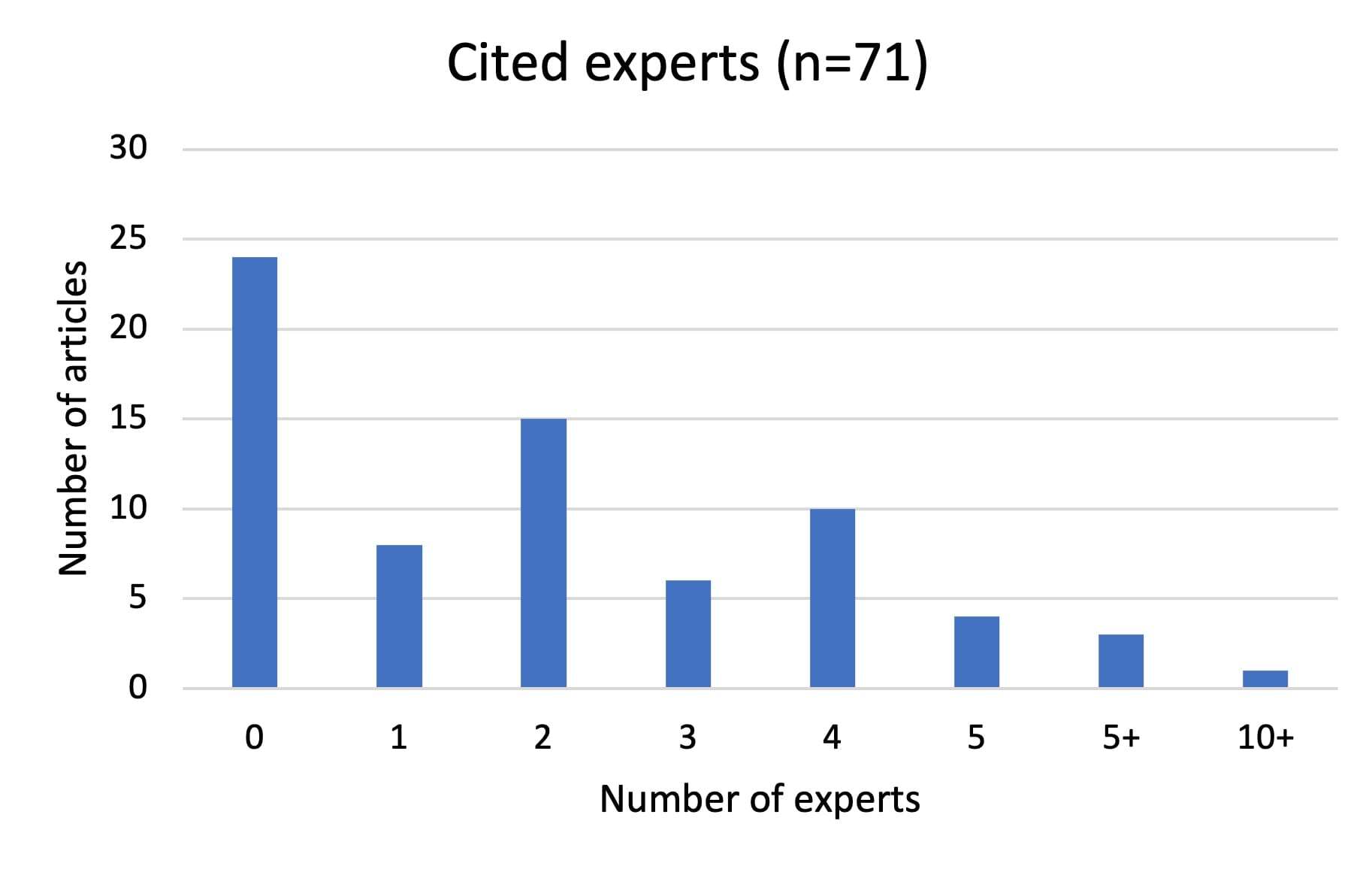


Supplemental Figure 7: Number of experts who are clearly quoted in the articles, shown as a bar chart


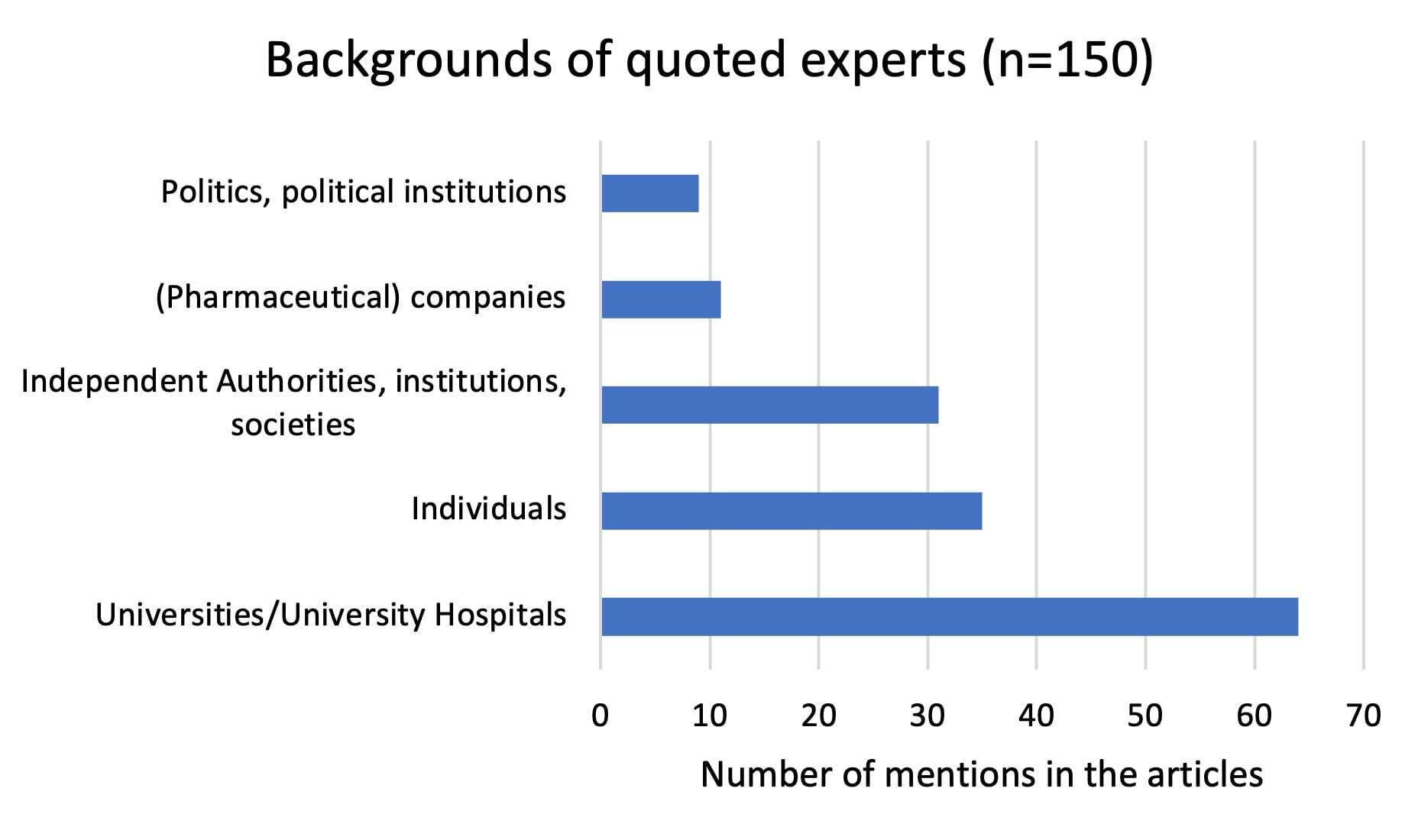


Supplemental Figure 8: Backgrounds of the experts quoted and/or named in the articles, shown as a bar chart


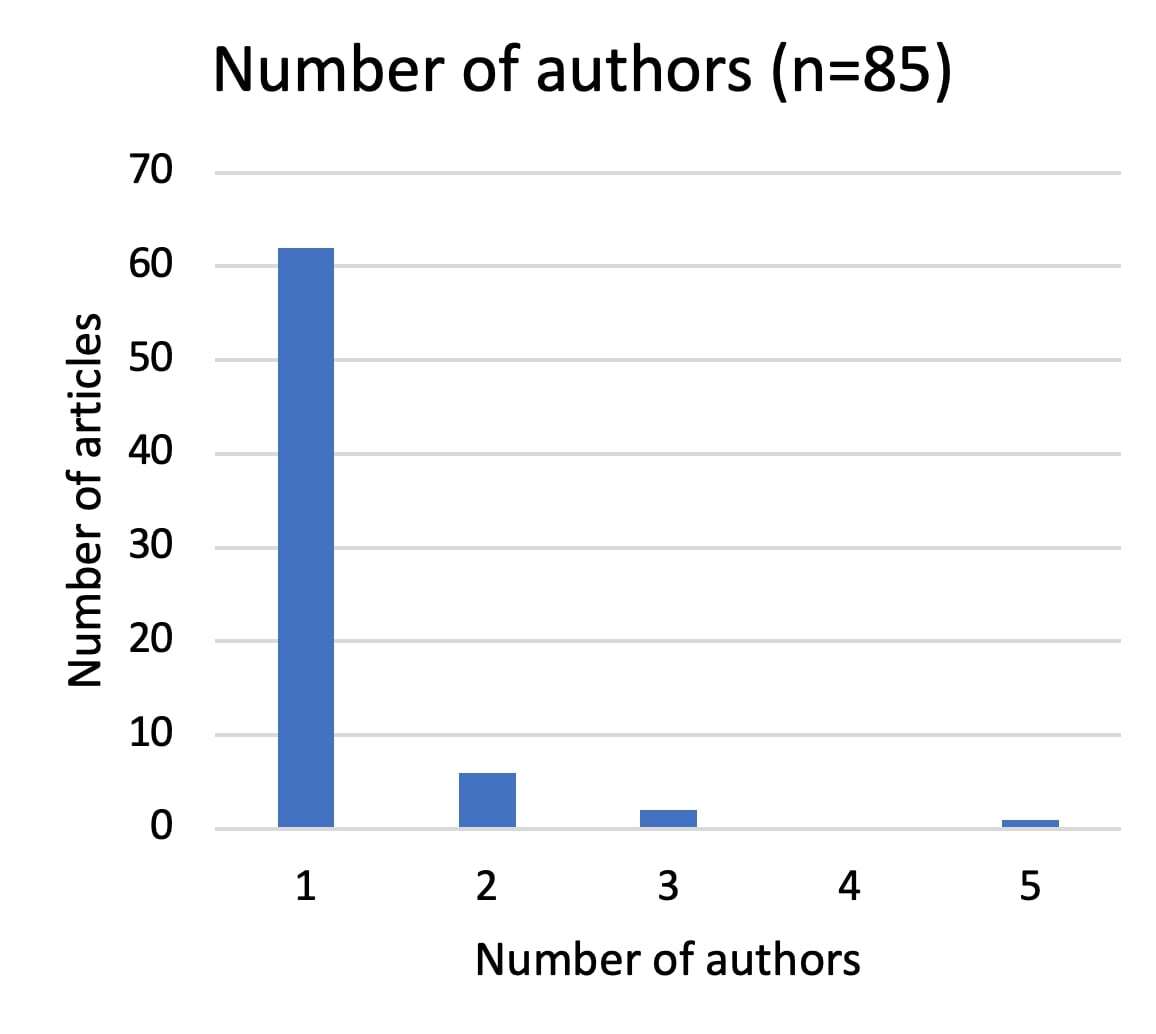


Supplemental Figure 9: Number of authors involved in the articles, shown as a bar chart


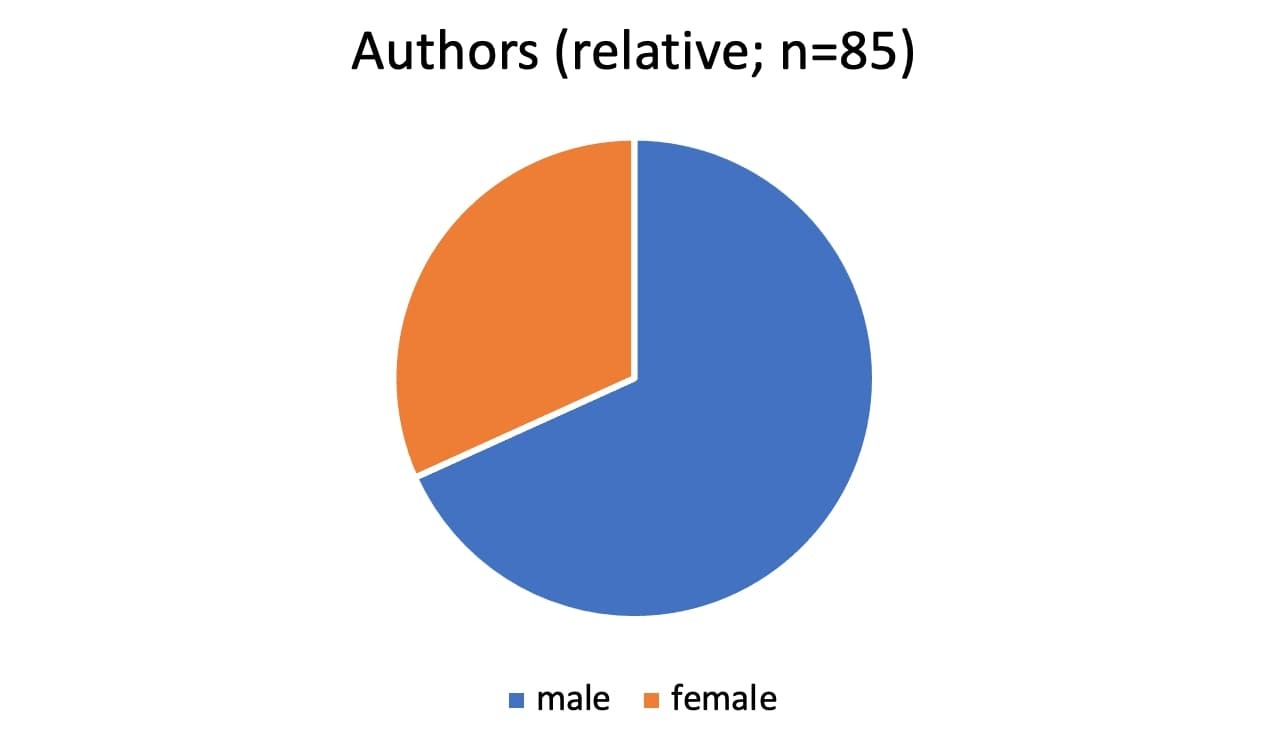


Supplemental Figure 10: Relative gender distribution of the 85 authors, shown as a pie chart


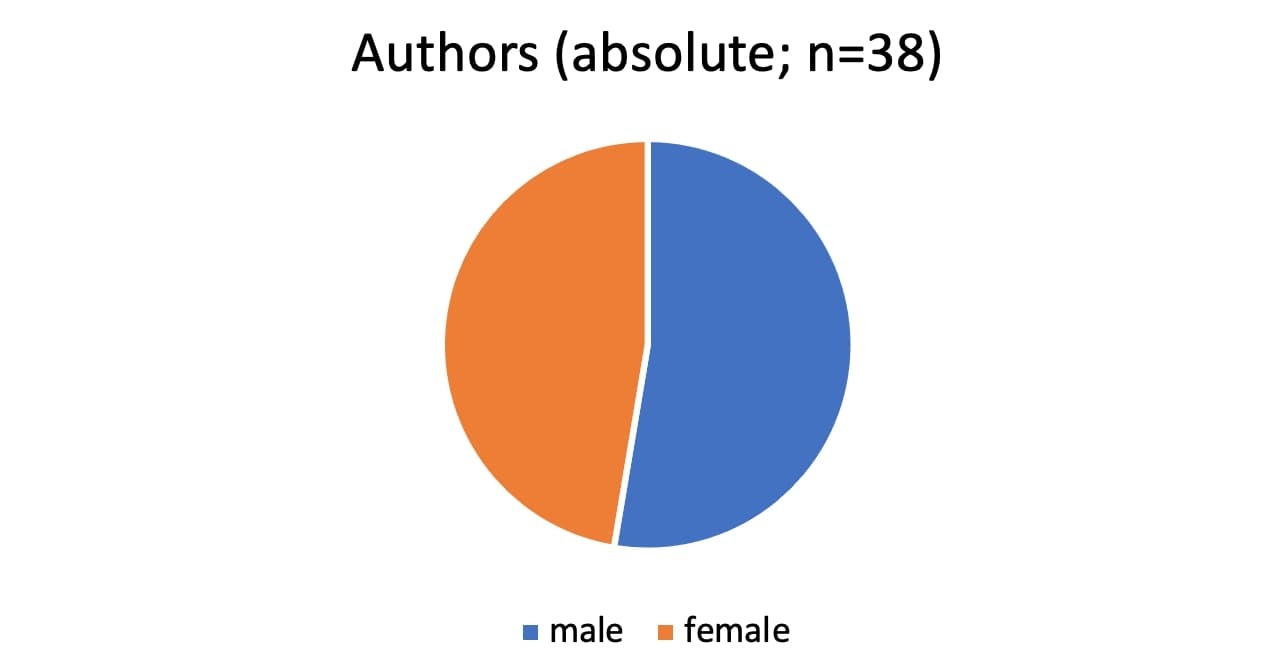


Supplemental Figure 11: Absolute gender distribution of the 38 authors, shown as a pie chart


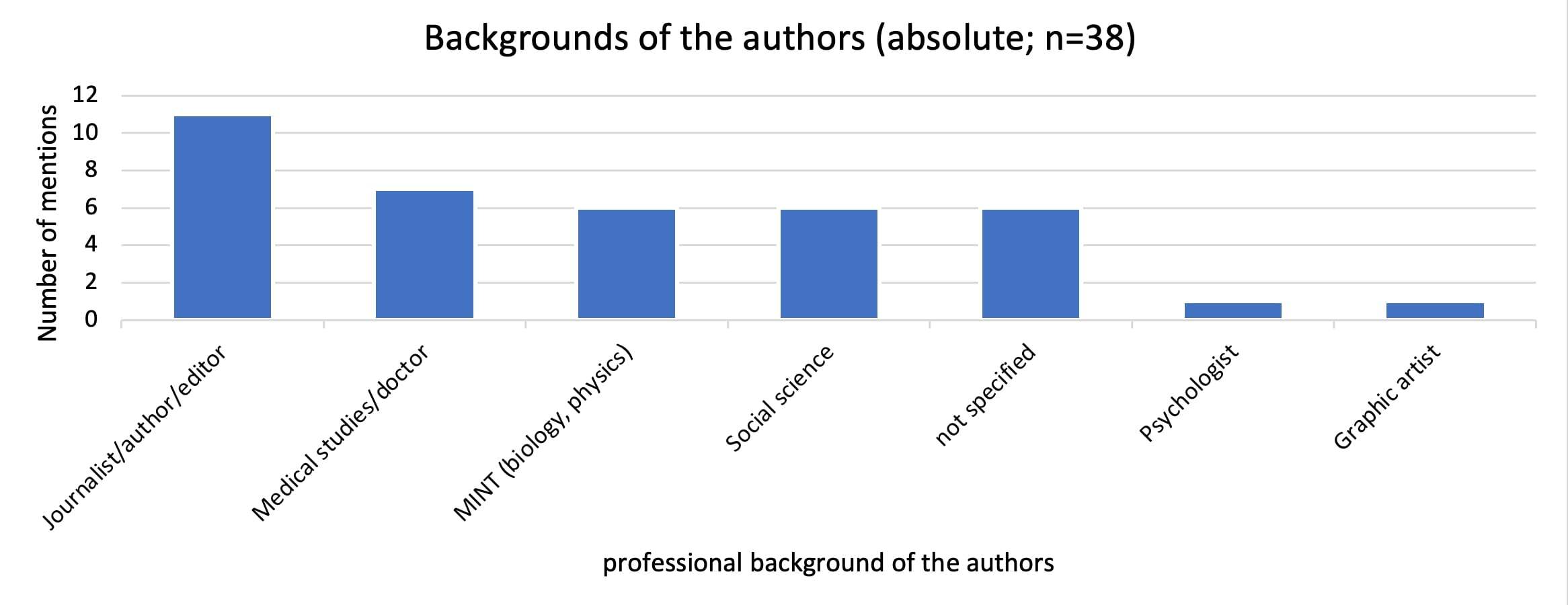


Supplemental Figure 12: Professional backgrounds of the authors, as listed on the ZEIT online page, shown as a bar chart


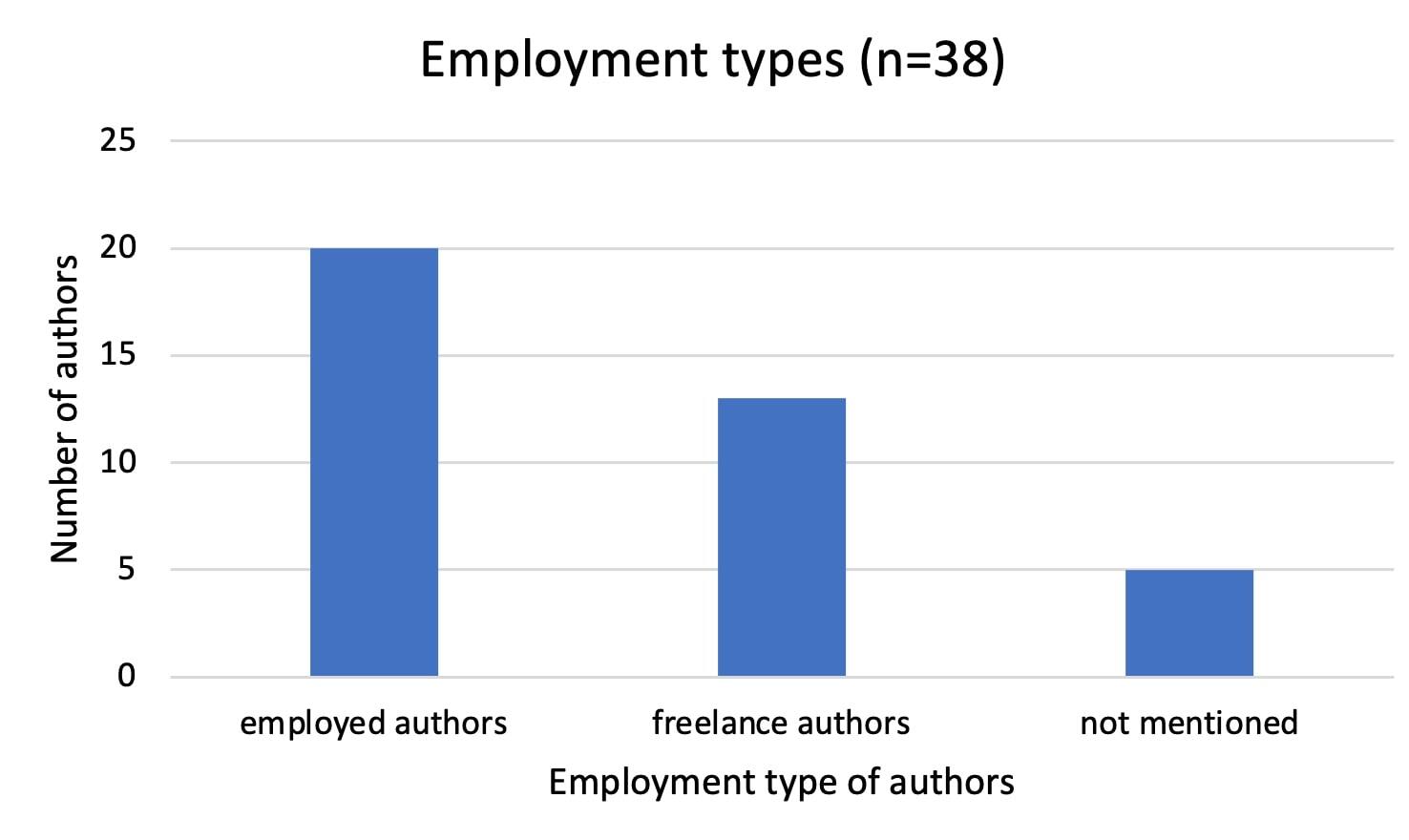


Supplemental Figure 13: Employment type of the authors, shown as a bar chart
